# Burnout and Perceived Stress Among Female Health Sciences Faculty Members at a University in Riyadh, Saudi Arabia: A Cross-Sectional Study

**DOI:** 10.3390/healthcare14193339

**Published:** 2026-10-07

**Authors:** Zeinab A. Abusabeib

**Affiliations:** Department of Community and Psychiatric Mental Health Nursing, College of Nursing, Princess Nourah bint Abdulrahman University, P.O. Box 84428, Riyadh 11671, Saudi Arabia; zaabusabeib@pnu.edu.sa

**Keywords:** burnout, perceived stress, health sciences faculty, female faculty, cross-sectional study, occupational health

## Abstract

**Highlights:**

**What are the main findings?**

- Emotional exhaustion was the most prominent dimension of burnout among faculty members across five health sciences colleges at a university in Riyadh.
- In multivariable analyses using continuous outcome scores, younger age and working more than 50 h per week were associated with higher emotional exhaustion and perceived stress.

**What are the implications of the main findings?**

- Faculty workload and career-stage characteristics warrant consideration in institutional well-being assessments.
- Future multicenter and intervention studies should evaluate whether organizational strategies targeting workload and faculty support can improve well-being.

**Abstract:**

**Background/Objectives:** Burnout and perceived stress are important concerns among health sciences faculty, whose roles often overlap across teaching, research, administrative, and clinical responsibilities. This study assessed burnout dimensions and perceived stress among female health sciences faculty at a single university in Riyadh, Saudi Arabia. **Methods:** This cross-sectional study included 228 female faculty members from five health sciences colleges, yielding a 64.4% response rate. Burnout was assessed using the Maslach Burnout Inventory–Educators Survey (MBI-ES), and perceived stress using the 10-item Perceived Stress Scale (PSS-10). Bivariate analyses incorporated effect sizes and false discovery rate adjustment for multiple comparisons. Multivariable linear regression models examined associations of demographic and professional characteristics with continuous emotional exhaustion and perceived stress scores. **Results:** For descriptive categorization, 34.2% of participants had high emotional exhaustion, 21.1% high depersonalization, 30.3% low personal accomplishment, and 15.2% high perceived stress. Perceived stress correlated most strongly with emotional exhaustion (r = 0.54, *p* < 0.001). In adjusted analyses, increasing age was associated with lower emotional exhaustion (B = −0.27, *p* < 0.001) and perceived stress (B = −0.16, *p* < 0.001), whereas working >50 h/week was associated with higher emotional exhaustion (B = 6.81, *p* < 0.001) and perceived stress (B = 3.13, *p* = 0.005). Overall adjusted college effects were not statistically significant. **Conclusion:** Longer working hours and younger age were associated with poorer faculty well-being, although the models explained modest proportions of outcome variance. These findings support institutional assessment of workload patterns and faculty well-being while underscoring the need for longitudinal research evaluating organizational demands, resources, and targeted interventions.

## 1. Introduction

Burnout is an occupational phenomenon characterized by emotional exhaustion, depersonalization or cynicism, and reduced professional accomplishment stemming from chronic workplace stress. Among university faculty, burnout and perceived stress are increasingly salient concerns because academic work entails multiple, often competing responsibilities, including teaching, research, student supervision, administrative duties, professional development, and, in health professions education, clinical or practice-based responsibilities. Sustained occupational stress may affect faculty psychological well-being and potentially influence professional functioning and the sustainability of academic work [1,2,3,4].

Faculty in health professions education may face a particularly complex mix of occupational demands. In addition to conventional academic responsibilities, some faculty members participate in clinical education, practical training, patient-related activities, and supervision in care-oriented settings. Evidence from academic and healthcare settings indicates that high workloads, prolonged hours, organizational pressure, and insufficient workplace resources may be associated with burnout and psychological distress. Although healthcare professionals in settings such as emergency care and other high-demand clinical environments differ substantially from university faculty, this broader occupational literature underscores the importance of considering both job demands and available organizational resources when examining professional well-being [5,6,7].

The Job Demands–Resources (JD-R) model offers a useful conceptual framework for this relationship. The model posits that sustained job demands requiring physical or psychological effort may contribute to strain, particularly when resources that facilitate work, recovery, professional development, and goal attainment do not adequately offset them. In health sciences academia, workload, teaching and clinical responsibilities, administrative duties, and competing scholarly expectations may constitute relevant job demands, whereas autonomy, organizational support, staffing, recognition, and professional resources may serve as job resources. The present study did not directly measure all of these demands and resources and therefore does not constitute a comprehensive empirical test of the JD-R model; rather, the framework contextualizes the workload- and career-related characteristics examined.

In Saudi Arabia, prior research has documented burnout and occupational stress among university faculty and health professions educators, with studies conducted within individual disciplines and across broader higher-education settings. However, studies vary substantially in disciplinary scope, measurement approaches, and the occupational characteristics assessed. Evidence examining burnout dimensions and perceived stress simultaneously among faculty from multiple health sciences disciplines operating within the same institutional environment is comparatively limited. Examining faculty across several health sciences colleges within one university does not provide nationally representative estimates, but it permits multidisciplinary assessment within a relatively shared organizational context [2,6,8,9,10].

Accordingly, this cross-sectional study aimed to assess burnout dimensions and perceived stress among female faculty members across five health sciences colleges at a single university in Riyadh, Saudi Arabia. The secondary objectives were to examine differences in emotional exhaustion and perceived stress by demographic and professional characteristics, evaluate correlations between burnout dimensions and perceived stress, and identify demographic and professional characteristics associated with emotional exhaustion and perceived stress when these outcomes were analyzed as continuous measures. We paid particular attention to workload- and career-related characteristics within the JD-R framework.

## 2. Materials and Methods

### 2.1. Study Design

A descriptive analytical cross-sectional design was used to assess burnout and perceived stress, as well as their associated factors among health sciences faculty members. An online self-administered questionnaire incorporating validated instruments was distributed electronically to promote broad participation and ensure confidentiality.

### 2.2. Study Area and Setting

The study was conducted at a prominent educational institution in Riyadh, Saudi Arabia, comprising five Colleges of Health Sciences: the College of Medicine, College of Dentistry, College of Pharmacy, College of Nursing, and College of Health Sciences and Rehabilitation. These colleges offer undergraduate and postgraduate programs, and faculty responsibilities include teaching, clinical practice, research, and administrative duties. All faculty members in the study population were female.

The standard academic workload for faculty members was approximately 35–40 h per week and included classroom teaching units and equivalent academic workload activities. Teaching responsibilities included undergraduate and graduate courses, graduation projects, field and clinical training, and supervision of master’s and doctoral theses. Equivalent workload activities included course preparation and assessment, academic advising, office hours, research activities, committee membership, coordination of multi-section courses, designated professional development activities, and electronic course development. The required classroom teaching load varied by academic rank, ranging from 10 h per week for professors to 16 h per week for lecturers.

Regarding employment arrangements, Saudi faculty members were employed under local permanent contracts, whereas non-Saudi faculty members were employed under annual contracts. Accordingly, contract type was closely tied to nationality and was not treated as a separate independent variable in the analysis.

### 2.3. Study Variables

The dependent variables were burnout and perceived stress. Burnout was assessed across three domains: emotional exhaustion, depersonalization, and personal accomplishment. Perceived stress was evaluated using the Perceived Stress Scale total score.

The independent variables included sociodemographic characteristics (age, nationality, marital status, and number of children) and professional characteristics (college affiliation, academic rank, years of teaching experience, weekly working hours, administrative role, involvement in practical or clinically oriented teaching, and research engagement).

Teaching experience and weekly working hours were collected as predefined categorical variables in the original questionnaire rather than as exact continuous values. Consequently, participant-level continuous values were not available for retrospective reanalysis. Teaching experience was recorded as <5, 5–10, and >10 years, and weekly working hours were recorded as <45, 45–50, and >50 h per week. These working-hour categories were investigator-defined at the questionnaire-design stage to reflect progressively greater weekly workload and were not derived from validated occupational thresholds.

### 2.4. Study Population

The target population comprised all full-time faculty members across the five Colleges of Health Sciences: Medicine, Dentistry, Pharmacy, Nursing, and Health Sciences and Rehabilitation.

### 2.5. Sampling Method and Sample Size

A census-based recruitment approach was used, and all eligible full-time faculty members across the five Colleges of Health Sciences were invited to participate. Eligibility criteria were applied before recruitment.

Inclusion criteria required that participants be full-time faculty members at any of the five Colleges of Health Sciences, have at least one year of teaching experience at the institution, and provide informed consent. Exclusion criteria included adjunct, part-time, and visiting faculty; administrative staff not directly involved in teaching; and faculty on extended leave, sabbatical, maternity leave, or medical leave during data collection.

### 2.6. Data Collection Techniques

Data collection took place during the first semester of the 2025–2026 academic year, from September 2025 to January 2026, using an online structured questionnaire with three sections. The first section collected demographic and professional information, including age, nationality, marital status, number of children, college affiliation, academic rank, years of teaching experience, weekly working hours, administrative responsibilities, involvement in practical or clinically oriented teaching, and research engagement.

The second section contained the Maslach Burnout Inventory–Educators Survey (MBI-ES), a 22-item instrument assessing burnout across three domains: emotional exhaustion, depersonalization, and personal accomplishment. The MBI-ES was used under license from Mind Garden, Inc., the authorized publisher and distributor of the instrument. Items are rated on a seven-point frequency scale from 0 (never) to 6 (every day). Higher emotional exhaustion and depersonalization scores, and lower personal accomplishment scores, indicate greater burnout.

The third section included the Perceived Stress Scale (PSS-10), a 10-item instrument that assesses perceived stress over the previous month. Items are rated on a five-point scale from 0 (never) to 4 (very often), yielding a total score from 0 to 40, with higher scores indicating greater perceived stress.

The questionnaire was administered in English, the primary academic language used across the participating health sciences colleges. The original validated English versions of the MBI-ES and PSS-10 were used without linguistic modification. The MBI-ES has been widely used among educators and university faculty, including health sciences faculty, and previous studies support its psychometric properties in academic populations. Previous studies have also used the MBI-ES with health sciences faculty in Saudi Arabia [11,12]. The PSS-10 is a widely used measure of perceived stress, and researchers have previously evaluated the psychometric properties of its English version among a Saudi university population [13]. In the present sample, we also assessed internal consistency for each MBI-ES domain and for the PSS-10. The online questionnaire was distributed through the designated faculty communication channels. No technical restrictions requiring login or limiting submissions to one response per participant were applied.

### 2.7. Data Processing and Analysis

Survey responses were exported from Google Forms (Google LLC, Mountain View, CA, USA; https://forms.google.com/, accessed on 6 October 2026) to Microsoft Excel (Microsoft Corporation, Redmond, WA, USA; https://www.microsoft.com/en-us/microsoft-365/excel, accessed on 6 October 2026) for initial data screening and preparation. Subsequent analyses were conducted using IBM SPSS Statistics, version 26 (IBM Corp., Armonk, NY, USA). Descriptive statistics summarized demographic and professional characteristics. Continuous variables were reported as means with standard deviations or as medians with interquartile ranges, as appropriate. Categorical variables were presented as frequencies and percentages.

Burnout domain scores were analyzed primarily as continuous variables. For descriptive purposes, MBI-ES domain scores were also categorized using the specified reference thresholds as follows: emotional exhaustion was classified as low (≤16), moderate (17–26), or high (≥27); depersonalization as low (≤6), moderate (7–12), or high (≥13); and personal accomplishment as low (≤31), moderate (32–38), or high (≥39) [14]. Because lower personal accomplishment scores indicate greater burnout, this domain was scored in the opposite direction of emotional exhaustion and depersonalization. These categories were used for descriptive purposes and were not interpreted as diagnostic thresholds.

For the PSS-10, the four positively worded items (4, 5, 7, and 8) were reverse-scored before summing all 10 items to obtain a total score ranging from 0 to 40, with higher scores indicating greater perceived stress. The PSS-10 total score was analyzed primarily as a continuous variable. For descriptive purposes and comparison with previous literature, PSS-10 scores were grouped as low (0–13), moderate (14–26), and high (27–40), consistent with categories commonly used in previous studies [15]. These categories were used only for descriptive presentation and were not interpreted as validated clinical or diagnostic thresholds. Internal consistency of the study instruments was assessed using Cronbach’s alpha. Continuous variables were summarized with means and standard deviations, and categorical variables with frequencies and percentages. Distributional assumptions for continuous outcomes were evaluated through graphical inspection, skewness and kurtosis, and the Shapiro–Wilk test. Homogeneity of variances across groups was assessed with Levene’s test. Given the approximately symmetric distributions of the continuous outcome scores and the sample size, parametric analyses were used where appropriate. Independent-samples *t*-tests were used for two-group comparisons, with Welch’s t-test applied when the homogeneity-of-variance assumption was violated. One-way analysis of variance (ANOVA) was used for comparisons involving three or more groups, with appropriate robust alternatives used when variance assumptions were not satisfied. Effect sizes and 95% confidence intervals were calculated alongside significance tests to quantify the magnitude and precision of observed differences.

Confidence intervals for effect-size estimates were obtained using bootstrap resampling. Given the number of bivariate comparisons, the Benjamini–Hochberg false discovery rate (FDR) procedure was applied to reduce the risk of type I error. Both unadjusted and FDR-adjusted *p*-values were considered when interpreting the exploratory bivariate analyses. Statistical significance was set at a two-sided *p*-value < 0.05.

Pearson correlation analysis was used to examine associations between burnout dimensions and perceived stress.

Multivariable linear regression analyses were conducted with emotional exhaustion and PSS-10 total scores as continuous dependent variables. Candidate explanatory variables were specified a priori based on theoretical relevance and the Job Demands–Resources framework, rather than selected solely on bivariate statistical significance.

Given the census-based recruitment design, no separate a priori sample-size calculation was used to determine the number invited to participate; all eligible faculty members were approached. The final analytical sample (*N* = 228) was considered adequate for the prespecified multivariable linear regression models relative to the number of estimated parameters, while the smaller sample sizes within some college categories were considered when interpreting college-specific coefficients.

College affiliation was entered as a categorical variable. Because the overall contribution of college affiliation was of primary interest, adjusted omnibus tests were used to evaluate the college variable as a whole. Individual college coefficients were treated as secondary pairwise contrasts and were not considered evidence of an overall college effect unless the omnibus test was statistically significant.

The models included age, college affiliation, weekly working hours, administrative role, practical/clinical teaching involvement, and research engagement. Age was entered as a continuous variable, whereas college affiliation and weekly working hours were modeled as categorical variables, with Pharmacy and <45 h/week as the reference categories, respectively. Administrative role, practical/clinical teaching involvement, and research engagement were entered as binary variables. Multicollinearity was assessed using variance inflation factors (VIFs), and no problematic multicollinearity was identified (all VIFs < 2.5). Regression coefficients (B), 95% confidence intervals, and *p*-values were reported for all variables entered into the models, regardless of statistical significance. Model performance was summarized using R^2^, adjusted R^2^, and the overall F-test.

The final analytical dataset contained no missing values for the variables included in the analyses; therefore, no imputation or other missing-data procedure was required.

### 2.8. Reporting Guideline

This study was reported in accordance with the Strengthening the Reporting of Observational Studies in Epidemiology (STROBE) guidelines for cross-sectional studies.

### 2.9. Ethical Considerations

The study was conducted in accordance with the Declaration of Helsinki. Ethical approval was obtained from the hosting university’s Institutional Review Board (IRB) (IRB Log No. 25-0518; approval date: 16 September 2025; KACST registration No. HAP-01-R-059). In accordance with the IRB approval conditions, the study site was not explicitly identified in the study setting. Participation was voluntary, and informed consent was obtained at the start of the online survey. Confidentiality and anonymity were maintained because no identifiable information was collected. All data were stored in password-protected files accessible only to the research team. Results were presented in aggregate, without disclosing the identities of individuals or departments.

## 3. Results

### 3.1. Response Rate and Participant Characteristics

**Figure 1:**
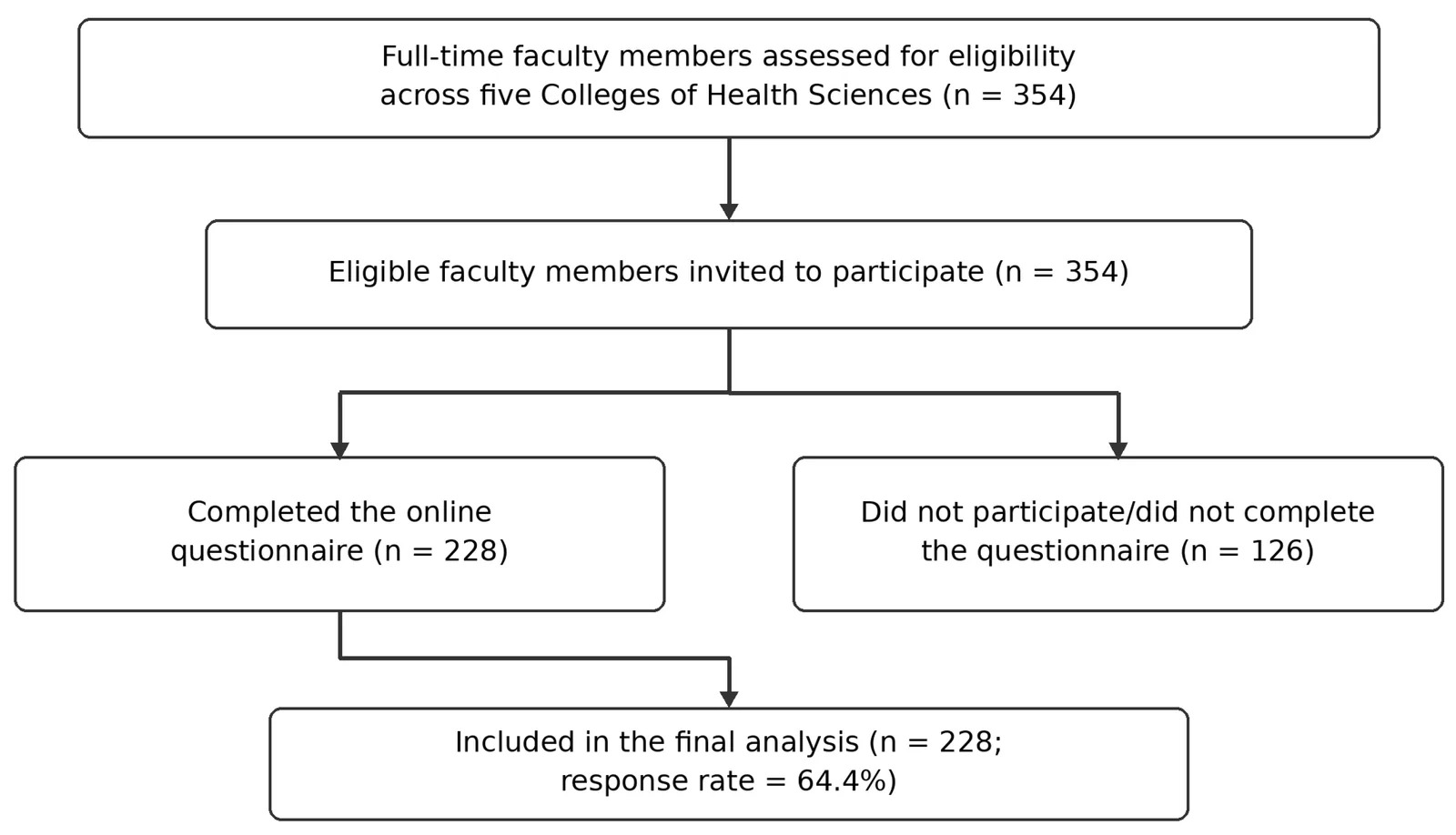
Participant recruitment and inclusion flow diagram.

Of the 354 eligible faculty members invited to participate, 228 completed the questionnaire, yielding a response rate of 64.4%. College-specific response rates were 70.8% for Medicine, 69.2% for Dentistry, 69.0% for Pharmacy, 67.4% for Nursing, and 55.8% for Health Sciences and Rehabilitation. Participants were distributed across the five Colleges of Health Sciences, with the largest share from Health Sciences and Rehabilitation, followed by Medicine and Dentistry. The mean age of respondents was 41.3 ± 8.9 years, and the majority were married and Saudi nationals. Assistant Professors were the largest academic rank group. Most participants had at least five years of teaching experience, and approximately one-fifth reported working more than 50 h per week. Detailed sociodemographic and professional characteristics are presented in Table 1. Participant recruitment and inclusion are summarized in Figure 1.

### 3.2. Internal Consistency of Study Instruments

The study instruments showed acceptable to excellent internal consistency, with Cronbach’s alpha ranging from 0.82 to 0.91 across the burnout domains and 0.86 for the PSS-10. The corresponding reliability coefficients are shown in Table 2.

### 3.3. Burnout and Perceived Stress Levels

The mean emotional exhaustion score was 22.6 ± 10.8. Approximately one-third of faculty members (34.2%) were classified as having high emotional exhaustion, while 39.0% and 26.8% were in the moderate and low categories, respectively. Low personal accomplishment, indicative of greater burnout in this domain, was reported by 30.3% of respondents.

The mean perceived stress score was 19.8 ± 6.2. Most faculty members reported moderate perceived stress, whereas 15.2% reported high perceived stress. Burnout and perceived stress levels are summarized in Table 3.

### 3.4. Differences in Emotional Exhaustion and Perceived Stress Across Demographic and Professional Characteristics

Differences in emotional exhaustion and perceived stress across demographic and professional characteristics are presented in Supplementary Table S1. In the unadjusted bivariate analyses, emotional exhaustion differed significantly by age group and weekly working hours, and perceived stress also differed significantly by age group and weekly working hours. After Benjamini–Hochberg false discovery rate (FDR) correction for multiple comparisons, only the associations of emotional exhaustion with age group and weekly working hours remained statistically significant.

Faculty under age 40 had higher emotional exhaustion scores than those aged 40 or older (25.0 ± 11.1 vs. 20.6 ± 10.1; unadjusted *p* = 0.002; FDR-adjusted *p* = 0.014; Cohen’s d = 0.41). Emotional exhaustion also differed across weekly working-hour categories (unadjusted *p* < 0.001; FDR-adjusted *p* = 0.013; η^2^ = 0.060), with the highest mean score among faculty working more than 50 h per week. The association between weekly working hours and perceived stress was statistically significant before FDR correction (unadjusted *p* = 0.012) but was no longer significant after correction. No other demographic or professional comparisons remained statistically significant after FDR adjustment.

### 3.5. Correlations Between Burnout Dimensions and Perceived Stress

Pearson correlation analysis showed that perceived stress was positively correlated with emotional exhaustion (r = 0.54, *p* < 0.001) and depersonalization (r = 0.37, *p* < 0.001), and negatively correlated with personal accomplishment (r = −0.31, *p* < 0.001). Emotional exhaustion showed the strongest correlation with perceived stress. Correlations among all study domains are presented in Table 4.

### 3.6. Multivariable Analysis of Emotional Exhaustion and Perceived Stress

Multivariable linear regression analyses were conducted with emotional exhaustion and PSS-10 scores as continuous outcomes (Table 5). In the emotional exhaustion model, each additional year of age was associated with lower emotional exhaustion scores (B = −0.27 per year; 95% CI: −0.42 to −0.12; *p* < 0.001), whereas working >50 h/week was associated with higher emotional exhaustion scores than working <45 h/week (B = 6.81; 95% CI: 3.09–10.53; *p* < 0.001). The overall adjusted association between college affiliation and emotional exhaustion was not statistically significant (omnibus F = 2.11, *p* = 0.081). The model explained 15.8% of the variance in emotional exhaustion (adjusted R^2^ = 0.119; overall model *p* < 0.001).

In the PSS-10 model, each additional year of age was associated with lower perceived stress scores (B = −0.16 per year; 95% CI: −0.25 to −0.07; *p* < 0.001), whereas working >50 h/week was associated with higher PSS-10 scores than working <45 h/week (B = 3.13; 95% CI: 0.94–5.33; *p* = 0.005). The overall adjusted association between college affiliation and PSS-10 score was not statistically significant (omnibus F = 1.20, *p* = 0.310). The model explained 10.8% of the variance in PSS-10 scores (adjusted R^2^ = 0.067; overall model *p* = 0.005). Other variables included in the models were not statistically significant. Complete estimates for all variables are presented in Table 5.

## 4. Discussion

This study examined burnout dimensions and perceived stress among female faculty members across five health sciences colleges at a single university in Riyadh. Emotional exhaustion was the most prominent burnout dimension, and perceived stress was moderately correlated with it.

In the adjusted analyses, increasing age was associated with lower emotional exhaustion and perceived stress scores, whereas working more than 50 h per week was associated with higher scores for both outcomes. In contrast, college affiliation was not significantly associated with either outcome when evaluated as an overall adjusted factor. These findings highlight workload and career-stage characteristics as relevant correlates of faculty well-being, while also indicating that the measured variables explained only a modest proportion of the observed variation.

The observed levels of burnout and perceived stress should be interpreted in the broader literature rather than as evidence of a uniquely high burden in this institution. Previous studies of university and health professions faculty have reported considerable variation in burnout estimates across populations, disciplines, measurement instruments, and classification criteria [3,16]. Accordingly, direct comparisons of prevalence percentages across studies should be made cautiously. The present findings contribute primarily by examining burnout dimensions and perceived stress simultaneously across several health sciences disciplines within a shared institutional environment, rather than by establishing new prevalence thresholds.

The positive correlation between perceived stress and emotional exhaustion aligns with the conceptual proximity of these constructs. Emotional exhaustion reflects the depletion of emotional and psychological resources related to work, whereas perceived stress reflects the extent to which individuals perceive circumstances as unpredictable, uncontrollable, or overwhelming [1]. Because this cross-sectional assessment is concurrent, it does not establish temporal direction, and the observed correlation should not be interpreted as evidence that one construct causes the other. This conceptual overlap also led to the exclusion of burnout dimensions as explanatory variables in the multivariable model of perceived stress.

Age showed an inverse association with both emotional exhaustion and perceived stress in the multivariable analyses. Similar patterns have been reported in parts of the faculty burnout literature, although age-related findings are not uniform across studies [12,17]. Potential explanations involving career development, autonomy, professional experience, or coping resources remain plausible but were not directly assessed in this study. The findings therefore support further investigation of career-stage differences rather than establishing younger age as a causal risk factor.

Weekly working hours showed the most consistent occupational association with the study outcomes. Faculty reporting more than 50 h per week had higher emotional exhaustion and perceived stress scores than those reporting fewer than 45 h per week. This pattern aligns with the Job Demands–Resources framework, which proposes that sustained job demands may contribute to strain when adequate resources for recovery, autonomy, support, and task completion are unavailable [18]. However, the working-hour categories in this study were investigator-defined and should not be interpreted as validated occupational thresholds. Furthermore, because of the cross-sectional design, the findings demonstrate an association with the highest predefined workload category rather than a causal effect of extended working hours.

The importance of workload and organizational resources is also evident in healthcare settings outside academia [19,20,21]. For example, research among nurses working in Italian prisons has documented burnout in a setting marked by distinctive occupational and organizational pressures, while research among Emergency Department nurses has demonstrated relationships between psychosocial job stress, working conditions, and health risks [22]. Although these professional environments differ substantially from those of university faculty, they illustrate a broader occupational health principle: persistent demands may be particularly relevant when combined with limited opportunities for recovery or insufficient organizational resources [8]. These comparisons should not be interpreted as indicating equivalence among prison nursing, emergency care, and academic employment; rather, they offer complementary evidence on the role of demanding work environments in professional well-being [20].

College affiliation requires careful interpretation. Although some individual college coefficients differed from the coding reference category, the overall adjusted omnibus tests for college affiliation were not statistically significant for either emotional exhaustion or perceived stress. Therefore, the present findings do not provide robust evidence of discipline-specific differences. The relatively small numbers within some colleges and the lack of detailed measures of discipline-specific workload, staffing, clinical responsibilities, and organizational resources further limit interpretation of contrasts among colleges. Future multicenter studies with larger discipline-specific samples are needed to determine whether meaningful differences exist across health professions disciplines.

Administrative responsibilities, practical or clinically oriented teaching, and research engagement were not independently associated with emotional exhaustion or perceived stress in the analyses [12,16]. These findings should not be interpreted as indicating that such responsibilities are irrelevant to faculty well-being. The measures used in this study captured participation in these activities rather than their intensity, complexity, protected time, or institutional support. A more detailed assessment of administrative workload, clinical supervision, research expectations, staffing, autonomy, and work–life balance may provide a more informative understanding of how academic responsibilities interact with faculty well-being.

### 4.1. Perspectives for Practice and Education in Healthcare Professionals

The findings have implications for how health sciences institutions assess faculty well-being, given that faculty responsibilities may include teaching, research, administration, student supervision, and, for some disciplines, clinical or care-related activities. Rather than interpreting burnout solely as an individual resilience issue, institutions may benefit from periodically assessing workload distribution, work-hour patterns, staffing, protected academic time, and access to organizational support [16].

The present study does not demonstrate that any specific intervention will reduce burnout; therefore, these strategies should be prioritized for institutional assessment and future intervention research.

This perspective aligns with occupational health evidence from other healthcare settings, where burnout, job stress, and related psychological outcomes have been linked to demanding work environments and organizational conditions [19,20,22]. Evidence on burnout and depression among healthcare workers further suggests that occupational well-being may extend beyond work satisfaction to encompass broader psychological health and professional functioning [19]. However, health sciences faculty occupy a distinct professional context, and clinical evidence should not be directly extrapolated to academic settings.

In health professions education, maintaining faculty well-being may be relevant to the sustainability of teaching, supervision, curriculum delivery, research, and clinical education. Institutions could therefore consider integrating faculty well-being indicators into broader workforce and educational quality assessments. Particular attention may be warranted to patterns of extended working hours and to career-stage differences identified through institutional monitoring. Longitudinal and intervention studies are needed to determine whether modifying specific job demands or strengthening organizational resources yields measurable improvements in faculty well-being.

### 4.2. Strengths and Limitations

This study has several strengths. The census-based recruitment strategy invited all eligible faculty members across five health sciences colleges and achieved an overall response rate of 64.4%. Examining multiple disciplines within the same university allowed faculty outcomes to be assessed in a relatively shared institutional environment. Established measures of burnout and perceived stress demonstrated good internal consistency in the study population. The analyses also treated emotional exhaustion and perceived stress primarily as continuous outcomes, reported effect sizes and confidence intervals, applied false discovery rate adjustment to multiple bivariate comparisons, and presented complete multivariable models.

Several limitations should be considered. The cross-sectional design precludes causal or temporal inference, particularly because burnout and perceived stress were assessed concurrently. Self-reported data may be affected by response and social-desirability biases. Although all eligible faculty members were invited, non-respondents may have differed from respondents, and response rates varied across colleges. The study was conducted at a single women-only university, limiting generalizability to male faculty, mixed-gender institutions, other universities, and other geographic settings. Sample sizes within individual colleges were also relatively small.

Data were collected from September 2025 to January 2026, spanning different phases of the academic semester. Burnout and perceived stress may fluctuate with teaching intensity, examinations, academic vacations, and administrative activities, but temporal variation within the data-collection period was not examined. The online survey did not include a technical restriction to prevent multiple submissions; therefore, duplicate responses could not be definitively excluded.

Teaching experience and weekly working hours were recorded as predefined categories rather than as exact continuous values, limiting the assessment of dose–response and nonlinear relationships. The working-hour categories were investigator-defined and should not be interpreted as validated occupational thresholds. In addition, several potentially important organizational factors—including leadership and organizational support, work–life balance, job satisfaction, organizational climate, staffing adequacy, autonomy, and detailed teaching and clinical workload—were not directly measured [16,23]. Consistent with these omissions, the multivariable models explained only a modest proportion of the variance in emotional exhaustion and perceived stress. Future multicenter longitudinal research incorporating detailed measures of job demands and resources is needed to clarify these relationships.

## 5. Conclusions

This cross-sectional study provides institution-specific evidence on burnout and perceived stress among female health sciences faculty members at a single university in Riyadh, Saudi Arabia. Increasing age was associated with lower emotional exhaustion and perceived stress scores, whereas working more than 50 h per week was associated with higher scores for both outcomes. College affiliation was not significantly associated with either outcome when evaluated as an overall adjusted factor. Given the modest explanatory power of the multivariable models and the cross-sectional design, these findings should not be interpreted as establishing causal or comprehensive determinants of faculty well-being. Institutional assessment of workload patterns and faculty well-being may help identify areas requiring support, while longitudinal and intervention studies that incorporate organizational resources, leadership, work–life balance, staffing, and detailed workload measures are needed to inform evidence-based strategies for health profession education.

## Figures and Tables

**Table 1:** Sociodemographic and professional characteristics of faculty respondents (*N* = 228).

Variable	Statistic/Category	*n*	%
Age (years)	Mean ± SD (range)	41.3 ± 8.9 (27–63)	—
Marital status	Single	53	23.2
	Married	159	69.7
	Divorced/widowed	16	7.1
Number of children	Median (IQR)	2 (1–3)	—
Nationality	Saudi	140	61.4
	Non-Saudi	88	38.6
College affiliation	Medicine	51	22.4
	Dentistry	45	19.7
	Pharmacy	29	12.7
	Nursing	31	13.6
	Health Sciences and Rehabilitation	72	31.6
Academic rank	Professor	3	1.3
	Associate Professor	63	27.6
	Assistant Professor	101	44.3
	Lecturer	44	19.3
	Instructor	17	7.5
Years of teaching experience	<5 years	66	28.9
	5–10 years	78	34.2
	>10 years	84	36.8
Weekly working hours	<45 h	89	39.0
	45–50 h	94	41.2
	>50 h	45	19.7
Administrative role	Yes	93	40.8
	No	135	59.2
Practical or clinically oriented teaching involvement	Yes	144	63.2
	No	84	36.8
Research engagement	Yes	178	78.1
	No	50	21.9

SD = standard deviation; IQR = interquartile range.

**Table 2:** Internal consistency of the MBI-ES and PSS-10.

Scale/Domain	Number of Items	Cronbach’s Alpha
Emotional exhaustion	9	0.91
Depersonalization	5	0.82
Personal accomplishment	8	0.88
Perceived Stress Scale	10	0.86

**Table 3:** Burnout and perceived stress levels among faculty members (*N* = 228).

Measure	Mean ± SD	Possible Range	Category	*n*	%
Emotional exhaustion (EE)	22.6 ± 10.8	0–54	Overall	228	100.0
			High (≥27)	78	34.2
			Moderate (17–26)	89	39.0
			Low (≤16)	61	26.8
Depersonalization (DP)	6.8 ± 5.1	0–30	Overall	228	100.0
			High (≥13)	48	21.1
			Moderate (7–12)	77	33.8
			Low (≤6)	103	45.2
Personal accomplishment (PA)	37.9 ± 7.4	0–48	Overall	228	100.0
			Low (≤31)	69	30.3
			Moderate (32–38)	95	41.7
			High (≥39)	64	28.0
Perceived Stress Scale (PSS-10)	19.8 ± 6.2	0–40	Overall	228	100.0
			Low (0–13)	48	21.1
			Moderate (14–26)	145	63.7
			High (27–40)	35	15.2

Note: For descriptive presentation, MBI-ES domain scores were grouped using the specified reference thresholds. These categories were not intended as diagnostic thresholds. PSS-10 categories were also used solely for descriptive presentation and were not intended as clinical or diagnostic thresholds.

**Table 4:** Correlations between burnout dimensions and perceived stress.

Measure	Emotional Exhaustion	Depersonalization	Personal Accomplishment	Perceived Stress
Emotional exhaustion	1.00	0.49 *	–0.42 *	0.54 *
Depersonalization	0.49 *	1.00	–0.35 *	0.37 *
Personal accomplishment	–0.42 *	–0.35 *	1.00	–0.31 *
Perceived stress	0.54 *	0.37 *	–0.31 *	1.00

Values represent Pearson’s correlation coefficients. * *p* < 0.001.

**Table 5:** Multivariable linear regression analyses of factors associated with emotional exhaustion and perceived stress scores.

Predictor	Category/Contrast	EE B (95% CI)	*p*-Value	PSS-10 B (95% CI)	*p*-Value
Age	Per 1-year increase	−0.27 (−0.42 to −0.12)	<0.001	−0.16 (−0.25 to −0.07)	<0.001
College	Pharmacy	Reference	—	Reference	—
	Medicine	4.97 (0.31 to 9.63)	0.037	2.03 (−0.73 to 4.78)	0.148
	Dentistry	3.68 (−1.10 to 8.45)	0.131	1.44 (−1.38 to 4.26)	0.314
	Nursing	6.43 (1.19 to 11.66)	0.016	3.11 (0.02 to 6.20)	0.049
	Health Sciences & Rehabilitation	2.01 (−2.40 to 6.42)	0.370	0.99 (−1.61 to 3.60)	0.454
Weekly working hours	<45 h/week	Reference	—	Reference	—
	45–50 h/week	2.57 (−0.43 to 5.57)	0.093	1.14 (−0.63 to 2.91)	0.206
	>50 h/week	6.81 (3.09 to 10.53)	<0.001	3.13 (0.94 to 5.33)	0.005
Administrative role	Yes vs. No	2.15 (−0.60 to 4.90)	0.125	0.56 (−1.06 to 2.19)	0.495
Practical/clinical teaching	Yes vs. No	1.85 (−0.97 to 4.66)	0.198	0.48 (−1.19 to 2.14)	0.573
Research engagement	Yes vs. No	−0.32 (−3.58 to 2.94)	0.846	−0.11 (−2.04 to 1.81)	0.910

Abbreviations: EE, emotional exhaustion; PSS-10, Perceived Stress Scale-10; B, unstandardized regression coefficient; CI, confidence interval. Pharmacy was used as the coding reference category for college affiliation, <45 h/week as the reference category for weekly working hours, and “No” as the reference category for binary variables. Overall adjusted omnibus tests for college affiliation were: EE, F = 2.11, *p* = 0.081; PSS-10, F = 1.20, *p* = 0.310. Model statistics: EE model, R^2^ = 0.158, adjusted R^2^ = 0.119, overall model *p* < 0.001; PSS-10 model, R^2^ = 0.108, adjusted R^2^ = 0.067, overall model *p* = 0.005.

## Data Availability

The data presented in this study are available on reasonable request from the corresponding author. The data are not publicly available due to privacy and ethical restrictions relating to the protection and confidentiality of human-participant data.

## Supplementary Materials

The following supporting information can be downloaded at: https://www.mdpi.com/article/10.3390/healthcare14193339/s1, Table S1: Emotional exhaustion and perceived stress scores by selected demographic and professional characteristics.

## Abbreviations

The following abbreviations are used in this manuscript:

DP	Depersonalization
EE	Emotional Exhaustion
MBI-ES	Maslach Burnout Inventory–Educators Survey
PA	Personal Accomplishment
PSS-10	Perceived Stress Scale-10
