# Supplementary material for: Burnout and Perceived Stress Among Female Health Sciences Faculty Members at a University in Riyadh, Saudi Arabia: A Cross-Sectional Study
Source: Healthcare (Basel). 2026 Oct 7;14(19):3339. doi: 10.3390/healthcare14193339 (PMC13665203; doi:10.3390/healthcare14193339)
Supplement: Supplementary file 1 [file healthcare-14-03339-s001.zip › healthcare-4550313-supplementary.pdf]

Supplementary Table S1. Emotional exhaustion and perceived stress scores by selected demographic and professional characteristics

Abbreviations: EE, emotional exhaustion; PSS-10, Perceived Stress Scale-10; SD, standard deviation; CI, confidence interval; FDR, false discovery rate. Cohen's d was used for two-group comparisons, and η^2 for comparisons involving three or more groups. FDR-adjusted p-values were computed using the Benjamini–Hochberg procedure for the 14 bivariate tests in the table. Bold values indicate statistical significance after FDR correction ($p < 0.05$).

Characteristic	Category	n	EE Mean ± SD	p-value	FDR p	Effect size (95% CI)	PSS-10 Mean ± SD	p-value	FDR-adjusted p	Effect size (95% CI)
**Age**	<40 years	104	25.0 ± 11.1	0.002	0.014	d = 0.41 (0.16–0.70)	20.8 ± 6.4	0.030	0.105	d = 0.29 (0.02–0.56)
	≥40 years	124	20.6 ± 10.1				19.0 ± 6.0			
**Teaching experience**	<5 years	66	24.9 ± 11.0	0.054	0.127	η^2 = 0.026 (0.004–0.085)	20.4 ± 5.8	0.276	0.404	η^2 = 0.011 (0.001–0.059)
	5–10 years	78	22.8 ± 10.9				20.2 ± 6.8			
	>10 years	84	20.6 ± 10.3				18.9 ± 5.9			
**College**	Medicine	51	24.6 ± 11.1	0.113	0.226	η^2 = 0.033 (0.011–0.110)	20.5 ± 6.5	0.447	0.568	η^2 = 0.016 (0.005–0.082)
	Dentistry	45	22.8 ± 10.5				19.8 ± 6.1			
	Pharmacy	29	19.8 ± 9.8				18.6 ± 5.7			
	Nursing	31	25.4 ± 11.4				21.1 ± 6.6			
	Health Sciences & Rehabilitation	72	21.0 ± 10.6				19.2 ± 6.1			

**Weekly working hours**	<45 h/week	89	19.9 ± 9.8	<0.001	0.013	η^2 = 0.060 (0.016–0.148)	18.6 ± 5.8	0.012	0.054	η^2 = 0.039 (0.007–0.109)
	45–50 h/week	94	23.0 ± 10.5				19.9 ± 6.2			
	>50 h/week	45	27.2 ± 11.7				22.0 ± 6.6			
**Administrative role**	Yes	93	23.8 ± 11.0	0.151	0.265	d = 0.19 (−0.08–0.47)	20.1 ± 6.3	0.547	0.639	d = 0.08 (−0.18–0.34)
	No	135	21.7 ± 10.6				19.6 ± 6.1			
**Practical/clinical teaching**	Yes	144	23.7 ± 10.9	0.051	0.127	d = 0.27 (0.01–0.55)	20.1 ± 6.3	0.288	0.404	d = 0.15 (−0.12–0.41)
	No	84	20.8 ± 10.4				19.2 ± 6.0			
**Research engagement**	Yes	178	22.5 ± 10.7	0.701	0.701	d = −0.06 (−0.39–0.26)	19.7 ± 6.2	0.698	0.701	d = −0.06 (−0.38–0.26)
	No	50	23.1 ± 11.2				20.1 ± 6.4			
